# The Two-Component System RstA/RstB Regulates Expression of Multiple Efflux Pumps and Influences Anaerobic Nitrate Respiration in Pseudomonas fluorescens

**DOI:** 10.1128/mSystems.00911-21

**Published:** 2021-11-02

**Authors:** Di-Yin Li, Jian-Ting Han, Meng-Yuan Zhang, Xu Yan, Nannan Zhang, Honghua Ge, Zhiping Wang, Yong-Xing He

**Affiliations:** a Ministry of Education Key Laboratory of Cell Activities and Stress Adaptations, School of Life Sciences, Lanzhou University grid.411294.bgrid.32566.34 , Lanzhou, P.R. China; b Institute of Urology, Lanzhou University Second Hospital, Key Laboratory of Urological Diseases in Gansu Province, Gansu Nephro-Urological Clinical Center, Lanzhou, P.R. China; c School of Life Sciences, Anhui University, Hefei, P.R. China; University of Illinois at Chicago

**Keywords:** two-component system, efflux pumps, multidrug resistance, *Pseudomonas fluorescens*

## Abstract

Multidrug resistance (MDR) efflux pumps are involved in bacterial intrinsic resistance to multiple antimicrobials. Expression of MDR efflux pumps can be either constitutive or transiently induced by various environmental signals, which are typically perceived by bacterial two-component systems (TCSs) and relayed to the bacterial nucleoid, where gene expression is modulated for niche adaptation. Here, we demonstrate that RstA/RstB, a TCS previously shown to control acid-induced and biofilm-related genes in Escherichia coli, confers resistance to multiple antibiotics in Pseudomonas fluorescens by directly regulating the MDR efflux pumps EmhABC and MexCD-OprJ. Moreover, we show that phosphorylation of the conserved Asp52 residue in RstA greatly enhances RstA-DNA interaction, and regulation of the multidrug resistance by RstA/RstB is dependent on the phosphorylation of the RstA Asp52 residue by RstB. Proteome analysis reveals RstA/RstB also positively regulates the efflux pump MexEF-OprN and enzymes involved in anaerobic nitrate respiration and pyoverdine biosynthesis. Our results suggest that, by coupling the expression of multiple efflux pumps and anaerobic nitrate respiration, RstA/RstB could play a role in defense against nitrosative stress caused by anaerobic nitrate respiration.

**IMPORTANCE** Microenvironmental hypoxia typically increases bacterial multidrug resistance by elevating expression of multidrug efflux pumps, but the precise mechanism is currently not well understood. Here, we showed that the two-component system RstA/RstB not only positively regulated expression of several efflux pumps involved in multidrug resistance, but also promoted expression of enzymes involved in anaerobic nitrate respiration and pyoverdine biosynthesis. These results suggested that, by upregulating expression of efflux pumps and pyoverdine biosynthesis-related enzymes, RstA/RstB could play a role in promoting bacterial tolerance to hypoxia by providing protection against nitrosative stress.

## INTRODUCTION

Rapidly sensing and responding to environmental stimuli is essential for bacteria to survive in constantly changing environments. Two-component systems (TCSs), which are ubiquitously distributed in prokaryotic genomes, play a pivotal role in bacterial sensing and responding to changes in their environment (1). A TCS comprises a sensor kinase and a response regulator, which are usually encoded adjacently in genomes (2, 3). A typical sensor kinase is composed of an N-terminal sensor domain facing the extracellular or periplasmic space and a C-terminal histidine kinase domain located in the cytosol (4). These two domains are connected by a transmembrane region which differs considerably in various TCSs (4). When exposed to environmental stimuli, the sensor domain undergoes drastic conformational changes, which are transmitted to the cytosolic histidine kinase domain through the transmembrane region, leading to auto-phosphorylation of a conserved C-terminal histidine residue in *trans* (5, 6). The phosphoryl group of the histidine residue is then transferred to a conserved aspartate residue of the responsive regulator, leading to its activation and transcription of downstream genes involved in diverse physiological processes such as chemotactic behavior, osmotic regulation, and virulence (7).

The prevalence of antibiotic-resistance in human pathogens has posed a great challenge to the treatment of bacterial infections (8, 9). Bacteria not only can be intrinsically resistant to certain antibiotics, but also acquire antibiotic resistance via mutations or horizontal gene transfers of antibiotic resistance genes (10). Three major mechanisms are responsible for antibiotic resistance: modification of drug targets, inactivation of antibiotics, and minimization of the intracellular drug concentrations (11). By extruding intracellular antibiotics as well as a wide range of other toxic compounds including heavy metals, solvents, and detergents, the transmembrane efflux pumps play an important role in antibiotic resistance (8, 12). Since aberrant expression of efflux pumps is generally associated with a fitness cost (13), their expressions must be tightly regulated in certain bacterial species, in which efflux pumps were found to be integrated in the complex regulatory networks of TCSs. For instance, Kunihiko et al. reported that over-expression of the responsive regulator component EvgA of the TCS EvgA/EvgS activates the EmrKY efflux pump and accounts for deoxycholate resistance in Escherichia coli (14). In Salmonella, the TCS BaeS/BaeR was demonstrated to regulate MdtABC and AcrD efflux pumps, which are related with the resistance to novobiocin and β-lactams antibiotics (15). Most recently, a number of other TCSs including AdeS/AdeR (16), CzcR/CzcS (17), AmgR/AmgS (18), and ParS/ParR (19) were found to be involved in regulating efflux pumps of antibiotics, highlighting the important role of TCSs in antibiotic resistance.

Pseudomonas fluorescens is a widely distributed environmental microbe which produces a variety of antibiotics through secondary metabolism to antagonize other competing bacteria and fungi. It also includes virulent strains involved in opportunistic infections (20–23). As P. fluorescens can be resistant to a wide spectrum of antibiotics (24), it is therefore urgent to better understand the multidrug-resistance mechanism of P. fluorescens. In P. fluorescens 2P24, the RND (resistance-nodulation-cell division) superfamily efflux pump EmhABC is characterized to be a key determinant to the resistance toward multiple antibiotics including ampicillin, chloramphenicol, and tetracycline (25, 26). However, it is still elusive if there are any other efflux pumps responsible for the multidrug resistance and how these efflux pumps are regulated in P. fluorescens. In this work, we demonstrate that RstA/RstB, a TCS previously shown to control acid-induced and biofilm-related genes in E. coli (27), confers resistance to multiple antibiotics in P. fluorescens by directly regulating the MDR efflux pumps EmhABC and MexCD-OprJ. Phosphorylation of the conserved Asp52 residue of RstA by RstB was shown be essential for the regulation function of RstA/RstB. Based on proteome analysis, we identify that RstA/RstB also positively regulated the efflux pump MexEF-OprN and enzymes involved in anaerobic nitrate respiration and pyoverdine biosynthesis, and propose a role for RstA/RstB in the cellular defense against nitrosative stress.

## RESULTS

### Cofitness analysis in P. fluorescens revealed a RstA/RstB-like TCS potentially involved in antibiotic resistance.

**FIG 1:**
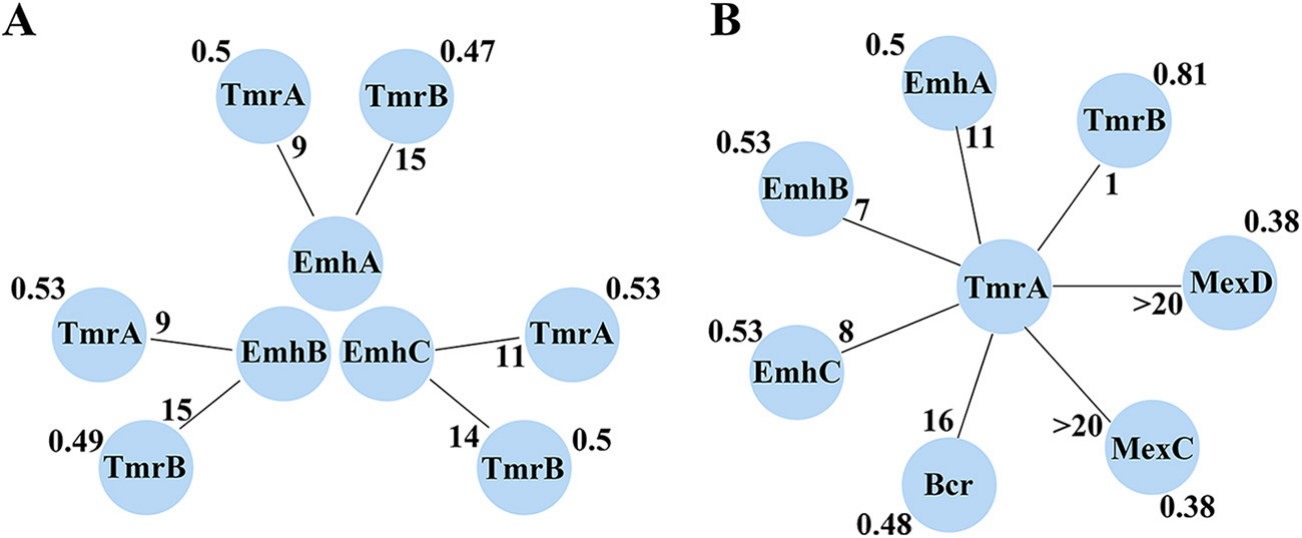
Cofitness values and ranks of (A) RstA/RstB and EmhABC, (B) RstA and other seven genes. The rank of each correlation is marked in the inner circle and cofitness values are marked on the outer ring.

The similarity of fitness under different growth conditions between any two different deletion strains has been defined as the cofitness (28), and two genes that have a high cofitness value tend to share similar biological functions. High-throughput data sets of microbial fitness had been proved to be informative in predicting gene functions and recently genome-wide mutant fitness data from 32 diverse bacteria across a set of growth conditions were available from http://fit.genomics.lbl.gov/, prompting us to seek the possible genes related to antibiotic resistance. In P. fluorescens, the protein products encoded by the *emhABC* operon were characterized to constitute an efflux pump playing a critical role in antibiotic resistance (25), we therefore reasoned that the strain lacking the resistance related genes should have high cofitness values with strains lacking the EmhABC efflux pump. Using the cofitness browser database, we analyzed the cofitness data of the mutant strains of P. fluorescens FW300-N2E2 with deletion of the genes *Pf6N2E2_2823*, *Pf6N2E2_2824*, and *Pf6N2E2_2825*, which are orthologues of *emhA*, *emhB* and *emhC*, respectively (∼98% sequence identity). Interestingly, two mutant strains with deletion of either *Pf6N2E2_463* or *Pf6N2E2_464* showed high cofitness values with above-mentioned three mutant strains (Fig. 1A), suggesting these two genes are likely to be related to antibiotic resistance. The genes *Pf6N2E2_463* and *Pf6N2E2_464*, respectively, encode a responsive regulator and sensor kinase of a TCS, which shares moderate sequence identities with the well-characterized RstA/RstB TCS (sequence identity 43%/35%) from E. coli. We next analyzed the cofitness data of Δ*Pf6N2E2_463* and found that three more mutant strains devoid of genes encoding other efflux pumps (*Pf6N2E2_1660*, *Pf6N2E2_1661*, *Pf6N2E2_3484*) have high cofitness values with the *ΔPf6N2E2_463* strain (Fig. 1B). The genes *Pf6N2E2_1660* and *Pf6N2E2_1661* are orthologues of the components of the MexCD efflux pump from Pseudomonas aeruginosa while *Pf6N2E2_3484* encodes a putative dipeptide transporter sharing 39% sequence identity with the bicyclomycin resistance protein Bcr in E. coli. Taken together, the cofitness analysis suggests that the RstA/RstB-like TCS (*Pf6N2E2_463* and *Pf6N2E2_464*) are likely to be involved in antibiotic resistance by regulating multiple efflux pumps including the EmhABC (*Pf6N2E2_2823*, *Pf6N2E2_2824*, and *Pf6N2E2_2825*), MexCD (*Pf6N2E2_1660*, Pf6N2E2_1661), and Bcr-like (Pf6N2E2_3484) pumps in P. fluorescens.

### The TCS RstA/RstB and MexCD-OprJ efflux pump contribute to antibiotic resistance in P. fluorescens 2P24.

To validate the potential antibiotic resistance related genes identified by cofitness analysis, we found the corresponding orthologues of these genes in the genome of P. fluorescens 2P24 (Table S3), constructed the single deletion mutants and measured the MICs of these mutants. *PFLP_02434* and *PFLP_02435* in P. fluorescens 2P24 correspond to *Pf6N2E2_463* and *Pf6N2E2_464*, respectively, and were named *rstA*/*rstB* as they encode proteins with the highest BLASTP score (43.85%/34.69% identity) with E. coli RstA/RstB proteins among the ORFs of P. fluorescens 2P24. Deletion of either of these two genes leads to increased susceptibility to multiple antibiotics including ampicillin, gentamicin, chloramphenicol, kanamycin, lomefloxacin, and tetracycline (Table 1). The *mexC* (*PFLP_03468*) deletion mutant, along with the *emhABC* deletion mutant, also showed decreased resistance to multiple drugs, yet with a slightly different antibiotic spectrum, suggesting different substrate specificities for these two efflux systems. The mutant devoid of the MFS transporter PFLP_00760, a homologous protein of Bcr, did not show significantly altered susceptibility to all the tested antibiotics (Table 1). Collectively, we identified the TCS RstA/RstB and the MexCD-OprJ efflux pump are involved in antibiotic resistance of P. fluorescens 2P24.

**TABLE 1:** Susceptibilities of the wild-type strain of P. fluorescens 2P24 and its constructed mutants to various antimicrobial agents

Strain	MIC (μg/mL)^*a*^					
	Amp	Chl	Kan	Tet	Gen	Lmf
Wild-type	512	128	4	8	2	0.5
*ΔrstA*	256	32	1	4	<0.25	<0.25
*ΔemhABC*	8	32	1	1	0.5	<0.125
*ΔmexC*	512	32	0.5	4	1	<0.25
*Δbcr*	512	64	4	16	4	1
*ΔrstB*-SD	256	128	2	4	1	0.5
*ΔrstB*-KD	256	32	2	4	0.5	<0.25
*rstA^D52A^*	512	16	2	4	0.5	<0.25
*rstA^D52E^*	256	16	0.5	2	<0.25	<0.25

a Amp, ampicillin; Chl, chloramphenicol; Kan, kanamycin; Tet, tetracycline; Lmf, lomefloxacin.

10.1128/mSystems.00911-21.2 TABLE S2 Primers used in this study. Download Table S2, DOCX file, 0.02 MB. Copyright © 2021 Li et al. 2021 Li et al. https://creativecommons.org/licenses/by/4.0/ This content is distributed under the terms of the Creative Commons Attribution 4.0 International license.

10.1128/mSystems.00911-21.3 TABLE S3 The genes in P. fluorescens FW300-N2E2 identified by cofitness and their respective orthologues in P. fluorescens 2P24 Table S3, DOCX file, 0.01 MB. Copyright © 2021 Li et al. 2021 Li et al. https://creativecommons.org/licenses/by/4.0/ This content is distributed under the terms of the Creative Commons Attribution 4.0 International license.

### RstA positively regulates the expression of *emhABC* and *mexCD-OprJ*.

**FIG 2:**
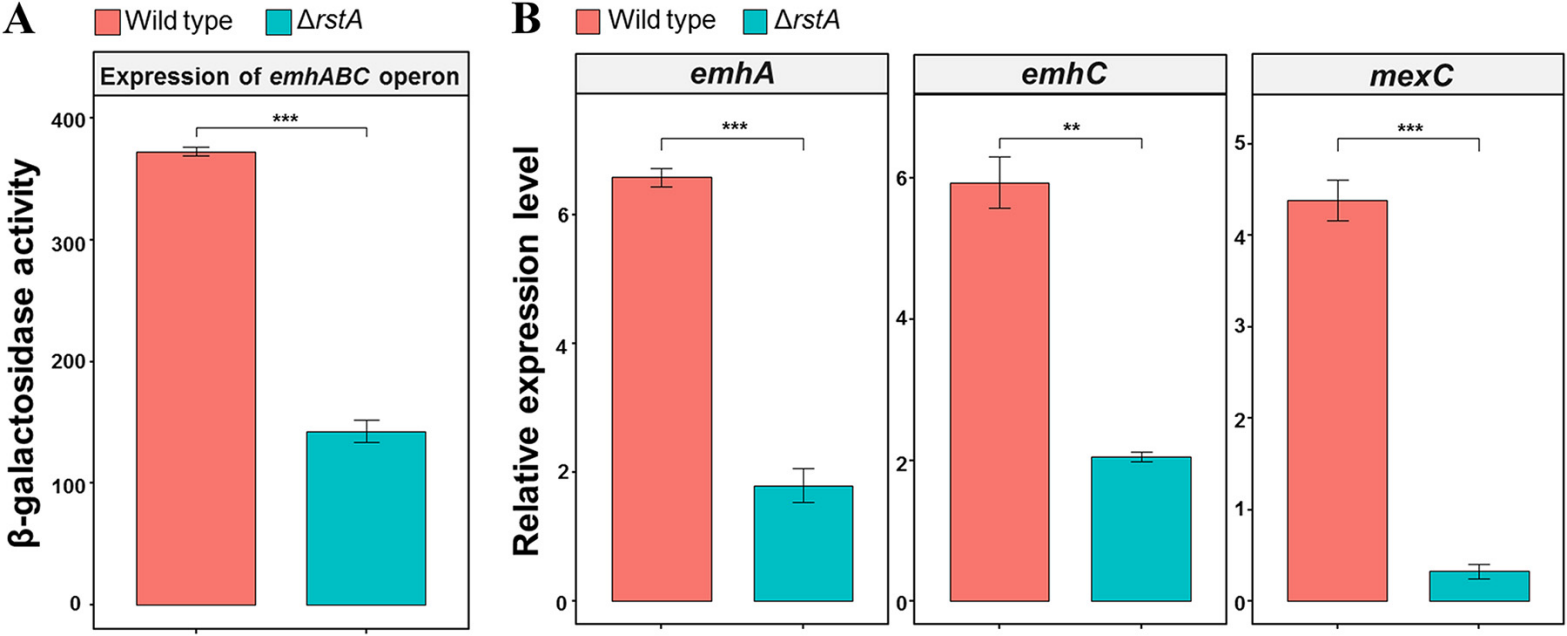
RstA positively regulates the expression of efflux pump *emhABC* and *mexCD*. (A) The β-galactosidase activities of wild-type strain and Δ*rstA*. (B)The transcription levels of *emhA*, *emhC* and *mexC* were measured in wild-type and Δ*rstA* strain via qRT-PCR assays. The expression level of 16S was used for normalization and 2^-ΔCt^ method was used for data analysis. Error bars denote standard deviation (*n* = 3). *P *less than * *0.01 was displayed as **, *P *less than .05 was displayed as *.

Given that the TCS RstA/RstB was shown to be related to antibiotic resistance, we asked whether the RstA regulator controls the expression of the multidrug efflux pumps EmhABC and MexCD-OprJ. Using the *lacZ* reporter fusion of the *emhA* promoter, we showed that expression of the *emhABC* operon was significantly decreased in the *rstA* deletion strain (Δ*rstA*) compared with that of the wild-type strain (Fig. 2A). Consistently, qRT-PCR assay revealed that deletion of *rstA* led to more than a 3-fold decrease of the *emhA* and *emhC* transcription levels (Fig. 2B). Moreover, by using qRT-PCR, we found that the Δ*rstA* strain showed a 10-fold decrease in *mexC* transcription level compared with the wildtype strain (Fig. 2B). Taken together, our results demonstrated that the responsive regulator RstA activates the expression of efflux pumps EmhABC and MexCD-OprJ in P. fluorescens strain 2P24.

### RstA binds to the promoter of *emhABC* and *mexCD-OprJ* in a phosphorylation dependent manner.

**FIG 3:**
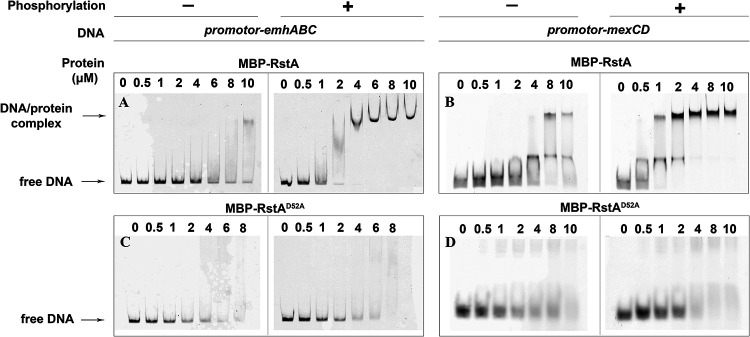
The EMSAs were used to determine the interaction between promoter-*emhABC* and RstA, phosphorylated-RstA (A, B), RstA^D52A^ or phosphorylated-RstA^D52A^ (C, D). The binding between promoter-*mexC* and RstA, phosphorylated-RstA, RstA^D52A^, or phosphorylated-RstA^D52A^ was determined with the same methodology. The concentrations of protein were labeled at the top of each lane. The protein phosphorylation state was indicated with + or –, respectively.

Given the regulatory role of RstA on EmhABC and MexCD-OprJ, we asked whether the RstA protein could directly interact with the promoter regions of the *emhABC* and *mexCD-OprJ* operons. The 265-bp upstream sequence of *emhA* was amplified and it was shown by using the electrophoretic mobility shift assays (EMSAs) that this DNA sequence weakly interact with RstA fused with an N-terminal maltose binding protein tag (MBP-RstA). As the DNA-binding ability of responsive regulators is generally dependent on phosphorylation of a conserved Asp residue, we performed an *in vitro* phosphorylation assay in which the MBP-RstA protein was incubated with acetyl phosphate, a high-energy phosphoryl-donor known to phosphorylate the conserved Asp residues in responsive regulators (29). Notably, the phosphorylated MBP-RstA (MBP-RstA^P^) interacted with the upstream sequence of *emh*A in a concentration dependent manner as shown by the EMSA (Fig. 3A). Moreover, phosphorylation of the MBP-RstA protein by acetyl phosphate also significantly enhanced its binding affinity toward the 101-bp upstream region of *mexCD-OprJ* (Fig. 3B). The appearance of two shifted bands for the *mexC* promoter suggested the presence of two RstA-binding sites, one with high binding affinity and another with low binding affinity. We also showed that MBP did not interact with the DNA probes used in the EMSAs (Fig. S1), excluding the possibility that MBP could interfere with the DNA-binding assays of RstA.

10.1128/mSystems.00911-21.5 FIG S1 EMSAs of MBP-tag or phosphorylated MBP-tag protein with *promoter-emhABC*. Promotors were incubated with different amounts of MBP-tag proteins (0 to 10 μM) in total reaction mixtures of 20 μl each. Download FIG S1, TIF file, 1.0 MB. Copyright © 2021 Li et al. 2021 Li et al. https://creativecommons.org/licenses/by/4.0/ This content is distributed under the terms of the Creative Commons Attribution 4.0 International license.

Although we attempted to use LC-MS/MS to pinpoint the phosphorylation sites of the MBP-RstA protein upon treatment by acetyl phosphate, however, no phosphorylated Asp residues were detected (data not shown). This is most likely due to the labile nature of phosphorylated Asp, which has an extremely short half-life and was notoriously difficult to capture. We next performed a multiple sequence alignment and identified that the Asp52 residue of RstA was strictly conserved among responsive regulators from various bacterial species (Fig. S2). The corresponding Asp residues in responsive regulators, such as CusR from E. coli (30), AlgR (31) and CpxR (32) from P. aeruginosa, have been reported to receive the phosphor-group from their cognate sensor kinases. To confirm if the 52 Asp residue of RstA is essential for the DNA-binding capacity, we purified the MBP-fused D52A mutant protein of RstA (MBP-RstA^D52A^) and tested its DNA-binding capacity. It was revealed that, compared with its wild-type counterpart, MBP-RstA^D52A^ had significantly compromised DNA-binding affinities toward the upstream sequences of *emhABC* and *mexCD-oprJ* regardless of the acetyl phosphate treatment (Fig. 3C and D). These results suggest the 52 Asp residue of RstA is likely to be the phosphorylation site that modulate the RstA-DNA interactions.

10.1128/mSystems.00911-21.6 FIG S2 Multiple sequence alignment of RstA. The Asp52 residue of RstA is indicated by an arrowhead. Download FIG S2, TIF file, 0.5 MB. Copyright © 2021 Li et al. 2021 Li et al. https://creativecommons.org/licenses/by/4.0/ This content is distributed under the terms of the Creative Commons Attribution 4.0 International license.

### Phosphorylation of the Asp52 residue of RstA by the RstB kinase contributes to the antibiotic resistance of P. fluorescens 2P24.

**FIG 4:**
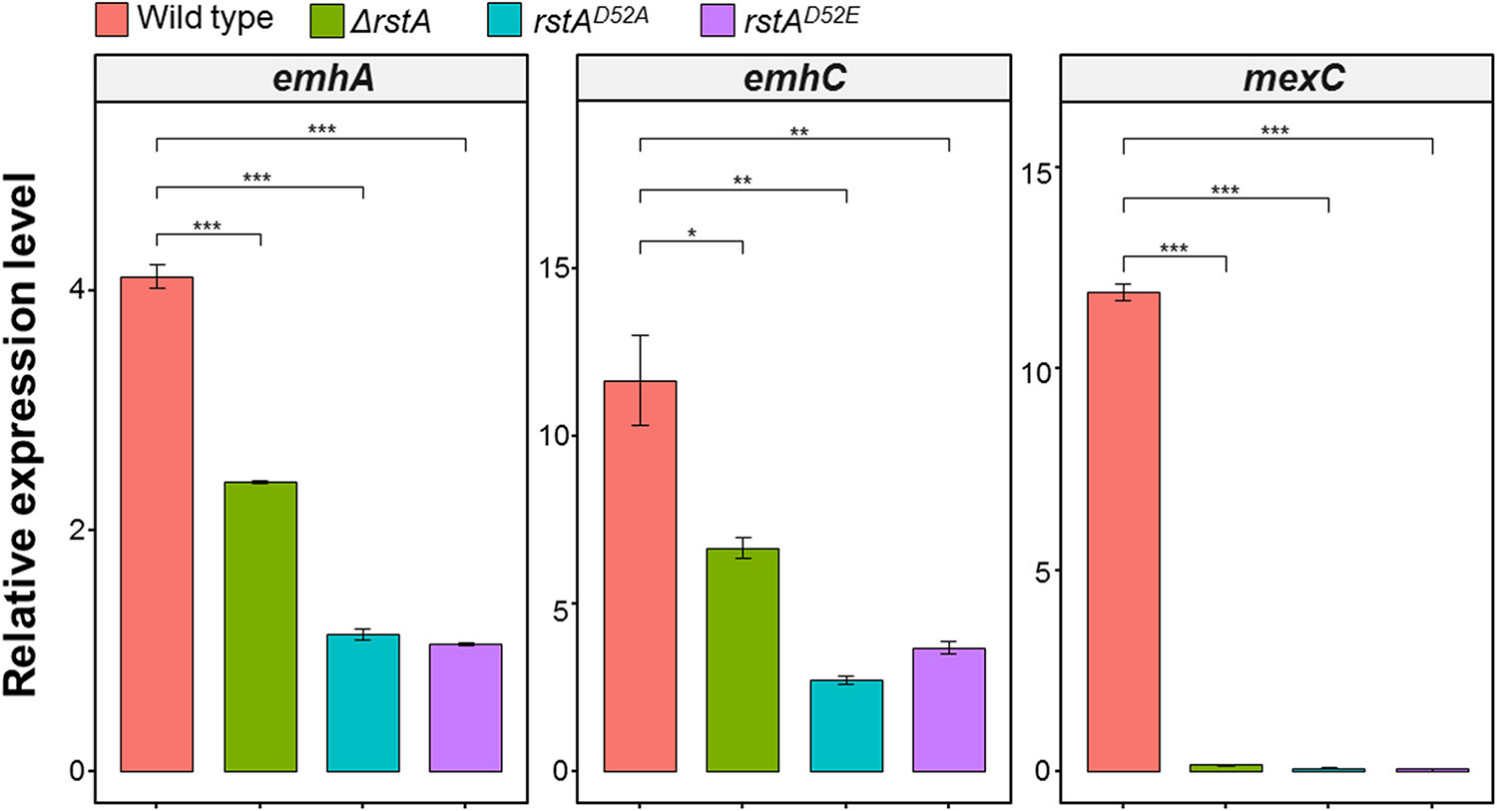
The qRT-PCR assays were employed to monitor the transcription levels of *emhA*, *emhC*, and *mexC* in different strains. The expression level of 16S was used for normalization and 2^-ΔCt^ method was used for data analysis. Error bars denote standard deviation (*n* = 3). *P *less than 0.001 was displayed as ***, *P * less than * *0.01 was displayed as **, *P *less than * *0.05 was displayed as *.

To investigate whether phosphorylation of RstA contributes to the antibiotic resistance of P. fluorescens 2P24, we constructed the mutant strain bearing the *rstA*^D52A^ single site mutation and tested its antibiotic resistance. Like the *ΔrstA* strain, this *rstA*^D52A^ mutant strain showed increased susceptibility to several antibiotics including chloramphenicol (8 folds), gentamicin (4 folds), kanamycin (2 folds), and lomefloxacin (>2 folds) (Table 1). Moreover, deletion of the *rstB-*kinase domain (Δ*rstB-*KD) also led to increased antibiotic susceptibility. Consistently, the expression levels of *emhA*, *emhC*, and *mexC* were significantly decreased in the *rstA*^D52A^, *rstA*^D52E^, and *ΔrstA* mutant strains compared with the wild-type strain (Fig. 4). It is interesting to note that *emhAC* expression is even less in the *rstA*^D52A^ strain than in the *rstA* deletion strain, suggesting that *rstA* may regulate some unknown factors that influence *emhAC* expression in a phosphorylation independent manner.

Interestingly, deletion of the sensor domain of *rstB* (*ΔrstB*-SD) had little effect on the antibiotic resistance (Table 1), implying either the intracellular kinase domain of RstB could be activated through cross-regulation by other TCSs or the ligands recognized by the sensor domain of RstB were absent in the culture medium. Taken together, our data indicates that phosphorylation of the Asp52 site of RstA by the sensor kinase RstB plays an important role in regulating antibiotic resistance.

### The responsive regulator RstA influences multiple physiological processes including nitrate respiration, pyoverdine biosynthesis, and drug efflux.

**FIG 5:**
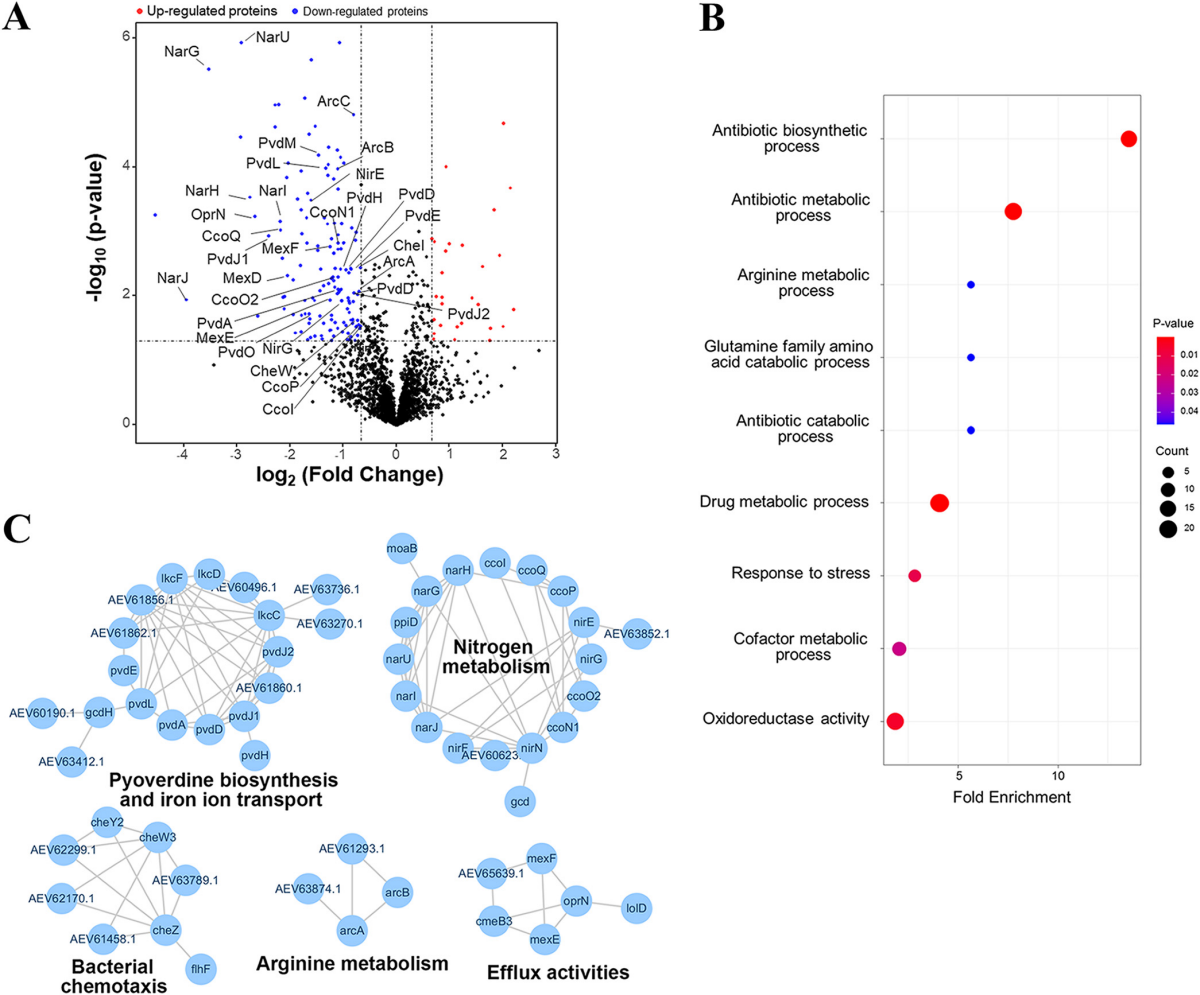
Comparative proteomic analysis of the wild-type and *RstA* mutant strains. (A) A volcano plot showing the differentially expressed genes (DEPs) in the Δ*RstA and D52A* strain compared with the wild-type strain of P. fluorescens 2P24. The red and blue dots indicate upregulated and downregulated genes, respectively, which are filtered based on the cut-off values of |log_2_FoldChange| > 1.5 and adjusted *P value*  less than 0.05. (B) Fisher’s extract test of RstA-affect proteins with the thresholds of *P value* less than 0.05 and enrichment factor greater than 1.5. The *P value* and protein count were represented with dot color and circle size. (C) STRING network analysis of DEPs in the *ΔrstA and rstA^D52A^* strain. Each node represents one protein and the interaction between proteins was displayed with lines.

In order to characterize the potential regulons of RstA in P. fluorescens 2P24, we performed label-free quantitative proteomics profiling of the wild-type and *ΔrstA* mutant strains. In total 2,354 proteins were identified with 1% false discovery rate (FDR), and hierarchical clustering using z-scored intensities of the 275 proteins quantified with high confidence (*P value* < 0.05) showed that four replicates of the samples of P. fluorescens and its derivatives *ΔrstA* were correctly clustered together (Fig. S3). Student's *t* test was used to screen differential expression proteins (DEPS) with fold change greater than 1.5 and *P value* less than 0.05 was used as thresholds, resulting in 33 upregulated proteins and 153 downregulated proteins (Fig. 5A). Gene ontology (GO) enrichment analysis revealed these DEPs were involved in many biological processes, especially antibiotic biosynthetic process, arginine metabolic process, and stress responses (Fig. 5B). To further study the biological pathways modulated by RstA, the interaction networks of downregulated proteins were created using the STRING database, showing that the downregulated proteins mainly participate in biological processes of pyoverdine biosynthesis and iron transport, nitrogen metabolism, bacterial chemotaxis, arginine metabolism, and efflux activities (Fig. 5C). Notably, several proteins including NarG1, NarG2, NarH, NarI, NarJ, NarU, NirE, NirF, NirG, and NirN involved in nitrate or nitrite reductase systems were markedly decreased in the *ΔrstA* strain (Fig. 5A). Moreover, proteins involved in pyoverdine biosynthesis including PvdA, PvdD, PvdE, PvdH, PvdL, PvdM, PvdO, PvdJ1, and PvdJ2 (33) were also significantly downregulated in the *ΔrstA* strain. The proteomic data also indicated that, in addition to the MexCD efflux pump, MexEF-OprN, an efflux pump that can extrude a variety of antibiotics as well as quorum sensing signals (34), was decreased in the *ΔrstA* strain. Collectively, our proteomic analysis demonstrated that the responsive regulator RstA not only activates the expression of several efflux pumps, but influences nitrate respiration and pyoverdine biosynthesis as well.

10.1128/mSystems.00911-21.7 FIG S3 Hierarchical clustering of the z-scored extracted ion chromatogram was used to evaluate the reproducibility of the proteome quantification between the wild-type and the Δ*rstA* strain. Download FIG S3, TIF file, 1.4 MB. Copyright © 2021 Li et al. 2021 Li et al. https://creativecommons.org/licenses/by/4.0/ This content is distributed under the terms of the Creative Commons Attribution 4.0 International license.

## DISCUSSION

Multidrug efflux pumps play an essential role in bacterial antibiotic resistance in both clinical and environmental settings (35, 36). The RND superfamily of efflux pumps, which are specific to Gram-negative bacteria and always form a tripartite complex spanning across the inner and outer membranes, are implicated in multidrug resistance in many bacterial species including the prominent human pathogens P. aeruginosa, E. coli, and Salmonella (37). In P. aeruginosa, there are several encoded RND-type efflux systems, four of which, i.e., MexAB-OprM, MexCD-OprJ, MexEF-OprN, and MexXY-OprM are characterized to be the significant determinants of multidrug resistance. Complex regulatory networks including multiple transcription regulators (MexR, MexT, NalC, NalD, NfxB, etc.) are involved in tight control of the expression levels of these efflux pumps. Disruption of the regulatory network that leads to upregulation of the efflux pumps have been commonly found in clinical isolates exhibiting the MDR phenotype. Being a close relative of P. aeruginosa, P. fluorescens produces a variety of antimicrobials and at the same time intrinsically resistant to multiple antibiotics, yet questions remain how the antibiotic resistance in P. fluorescens is regulated. Our work demonstrated that the TCS RstA/RstB contributes to antibiotic resistance of P. fluorescens by activating expression of the EmhABC and MexCD-OprJ efflux pumps, highlighting the important roles of certain TCSs in modulating intrinsic antibiotic resistance. In E. coli and Salmonella, RstA was reported to regulate genes related to biofilm formation and iron acquisition (27, 38), and the classic RstA binding motif (TACAN_6_TACA) was not found in the promoter regions of major antibiotic efflux pumps in these bacterial species (39). These results suggest an evolutionarily divergent role of RstA/RstB in regulating RND efflux pump expression among different bacterial species.

As bacteria are constantly exposed to different levels of antibiotics or toxic compounds, it is unsurprising that expression of efflux pumps could be integrated into the TCS regulatory network, which could transiently upregulate the efflux pumps in response to certain effectors or environmental stimuli. When a sensor kinase detects a specific signal, it usually undergoes auto-phosphorylation and subsequently transfer the phosphate group to its cognate responsive regulator, enabling genome-scale transcriptional activation by the responsive regulator (4). Consistently, we found that deletion of the cytosolic kinase domain of RstB leads to antibiotics susceptibility, confirming the essential role of the RstB kinase domain in activating the RstA responsive regulator. Sequence analysis indicates that RstA belongs to the OmpR/PhoB protein family, which comprises approximately 40% of all response regulators. Notably, the conserved D52 residue was pinpointed as the phosphorylation site modulating the DNA binding affinity of RstA and mutation of this aspartate into Ala leads to the decreased antibiotic resistance of P. fluorescens, highlighting that phosphorylation of D52 acts as a molecular switch governing the activity of RstA. It was previously shown that for the OmpR/PhoB family responsive regulators such as DrrB, DrrD (40), and MtrA (41), phosphorylation of the conserved aspartate residue promotes dimerization and brings the DNA-binding domains into proximity, resulting in the enhanced DNA-binding affinity. Collectively, our data support that a conserved phosphorylation-dependent regulation mechanism is shared among the responsive regulators of the OmpR/PhoB family.

What kind of signals the sensor domain of RstB detects is currently unknown. Interestingly, deletion of the RstB sensor domain barely affects the antibiotic resistance of P. fluorescens, suggesting that the histidine kinase domain of RstB could either be phosphorylated by other sensor kinases or undergo basal auto-phosphorylation without any extracellular signal input. By using the three-dimensional structure prediction server Phyre we found that the RstB extracellular domain is predicted to share a similar structural fold with the NarQ extracellular domain (42), despite the low sequence identity (∼23%). NarQ, together with NarX, are known to be sensor kinases that respond to nitrate and nitrite to regulate enzymes involved in anaerobic respiration and fermentation (43, 44). Moreover, our proteomic data clearly demonstrated that the responsive regulator RstA positively regulates the enzymes involved in anaerobic nitrate respiratory chain (NarG, NarH, NarI, NarJ, NarK) (45, 46) and arginine deiminase pathway (ArcA, ArcB, ArcC, ArcD) (47), which are typical regulons of NarQ. All these pieces of evidence direct to a possible role in sensing nitrate/nitrite/their analogues and regulating anaerobic nitrate respiration and arginine fermentation for the TCS RstA/RstB.

**FIG 6:**
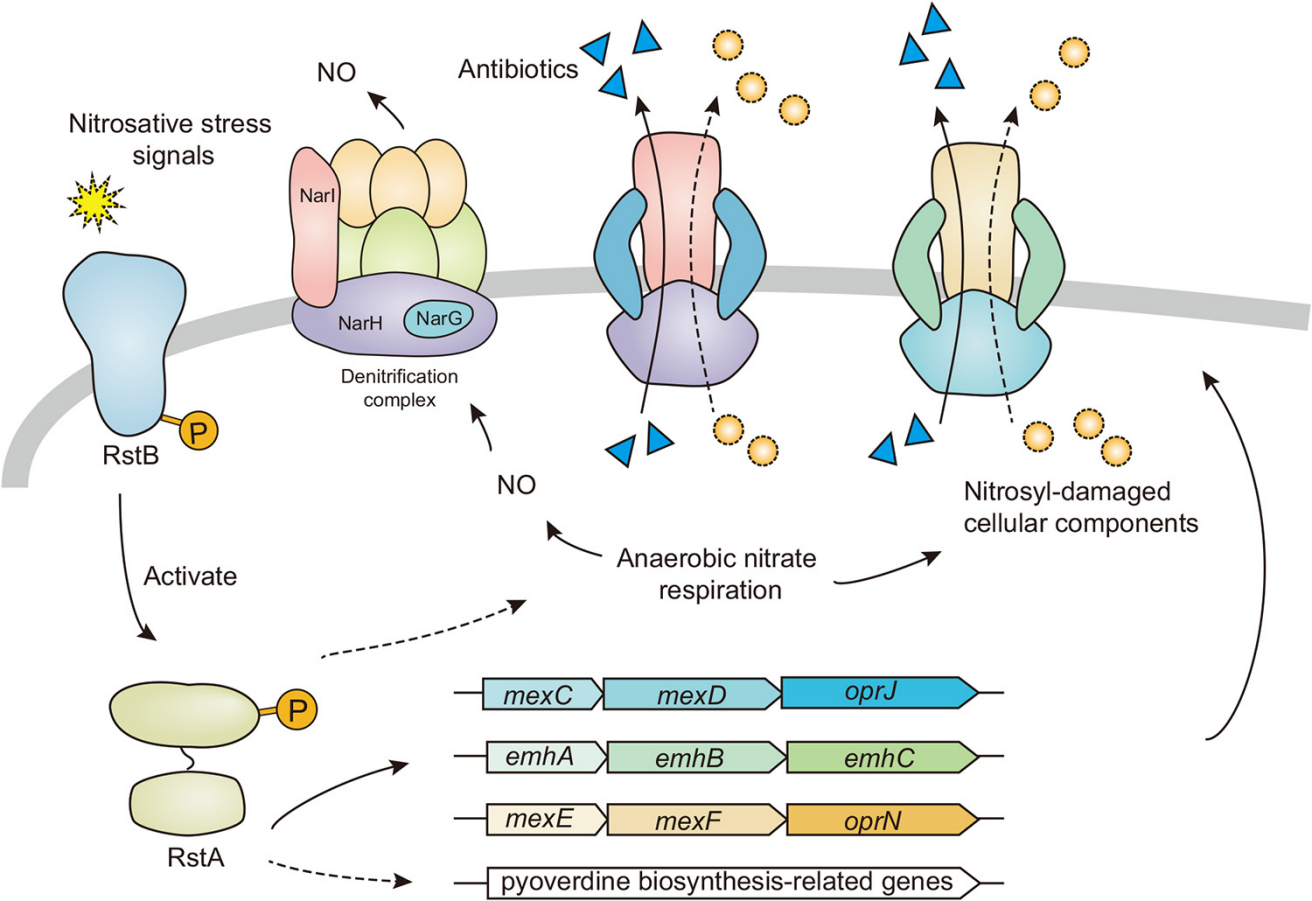
A working model for the physiological roles of RstA/RstB under nitrosative stress of P. fluorescens 2P24. During anaerobic nitrate respiration in P. fluorescens 2P24, RstB is activated by sensing relative signal molecules and activates RstA which upregulates expression of efflux pumps and pyoverdine biosynthesis-related enzymes. This process not only protects cells against nitrosative damage by extruding the nitrosyl-damaged cellular components but increases antibiotic resistance. The solid arrows denote direct regulations while the dashed arrows denote indirect regulations.

Several Pseudomonas species including P. fluorescens can perform denitrification under anaerobic conditions, using nitrate instead of oxygen as a terminal electron acceptor (48). This process, also known as anaerobic nitrate respiration, generally results in the formation of NO and/or other reactive nitrogen species which exert nitrosative stress on the cells (49). Therefore, the sensing of such compounds as well as the induction of nitrosative stress defense against proteins appears to be vital for cell proliferation. The multidrug efflux pump MdtEF-TolC in E. coli (50) and MexEF-OprN in P. aeruginosa (51) had been shown to be induced in response to nitrosative stress and protect against nitrosative damage by extruding the nitrosyl-damaged cellular components. Genes involved in the production of pyoverdine were also implied in resistance to nitrosative stress (52). It is therefore likely that RstA/RstB can function as a sensor for nitrosative stress, and upregulates expression of efflux pumps and pyoverdine biosynthesis-related enzymes to protect against nitrosative stress (Fig. 6). On the other hand, overexpression of efflux pumps leads to enhanced consumption of oxygen, causing microenvironmental hypoxia, which in turn promotes anaerobic nitrate respiratory in P. aeruginosa (13). It should be interesting to investigate how the TCS RstA/RstB could be involved in mediating the interplay between expression of efflux pumps, biosynthesis of pyoverdine and anaerobic nitrate respiration in the future.

## MATERIALS AND METHODS

### Bioinformatics analysis.

The homozygous cofitness data of Pf6N2E2_2823, Pf6N2E2_2824, Pf6N2E2_2825, Pf6N2E2_463, Pf6N2E2_464, Pf6N2E2_1660, Pf6N2E2_1661, and Pf6N2E2_3484 deletion strains were acquired from the online Cofitness Browser Database (http://fit.genomics.lbl.gov/). The sequence alignment was performed using the Basic Local Alignment Search Tool of the NCBI website and the Multalin page (http://multalin.toulouse.inra.fr/multalin/).

### Bacterial strains, plasmids, and culture conditions.

The bacterial strains and plasmids are listed in Table S1. E. coli strains were grown at 37°C in Luria-Bertani (LB) broth. P. fluorescens 2P24 was grown at 28°C in King’s B (KB) medium (31). Ampicillin and kanamycin was used at a concentration of 50 μg/ml.

10.1128/mSystems.00911-21.1 TABLE S1 Strains and plasmids used in this study. Download Table S1, DOCX file, 0.02 MB. Copyright © 2021 Li et al. 2021 Li et al. https://creativecommons.org/licenses/by/4.0/ This content is distributed under the terms of the Creative Commons Attribution 4.0 International license.

### Deletion of gene loci in P. fluorescens 2P24.

The in-frame deletion mutants were constructed via double-crossover homologous recombination with the suicide plasmid pK18Km as reported previously (26). For each gene, both flank regions (longer than 1Kb) including 100 bp in-frame were PCR-amplified, respectively, and linked together using overlap PCR. The linked fragment, cloned into pK18mobsacB, was transported into P. fluorescens 2P24 by conjugating with the E. coli strain WM3064 to replace the target gene locus. In brief, after transformed with the suicide plasmid, the WM3064 and the wild-type P. fluorescens 2P24 strains were mixed in the same dilution rates and plated on a KB agar plate for 6 h to 8 h. Following scraping little bacterial plaque in the double-stress (ampicillin, kanamycin) KB medium, proper dilutions were plated on a double-stress KB agar plate and single crossover strains were validated and cultured in the KB medium for 8 h. Under a 10% sucrose (wt/vol%) stress, mutant strains were confirmed by PCR amplification after the second crossover.

### MIC.

The MIC values were identified via a serial dilution method using 96-well cell culture plates which contained graded concentrations (0.25–512 μg/ml) of each antibiotic. Strains of log phase (OD600 = 0.8) were diluted to a final concentration of 10^5^/ml with KB broth and inoculated to each well. After incubated at 28°C for 20 h, the MIC readings of the wells were detected by a microplate reader at a wavelength of 600 nm (Absorbance Reader ELx808, BioTek) (53).

### Construction of LacZ fusion reporter plasmid and β-galactosidase assays.

We used a kanamycin resistance plasmid pRG970Km to construct *lacZ* fusions as previously reported (54). Purified 270 bp DNA fragments of the upstream sequence of *emhABC* and the plasmids were digested by BamHI and *CAIP*, after which the fragment was cloned ahead of a promoter-less *lacZ* gene. The plasmid transformation was performed via conjugating between a donor strain (E. coli WM3064) and a recipient strain (P. fluorescens 2P24) as mentioned above. As reported by Miller before (55), the LacZ expression levels were measured by β-galactosidase assays. Bacterial cultures were grown at 28°C in KB medium to the OD_600_ of 0.5 and the o-nitrophenol (Sigma-Aldrich, St. Louis, MO, USA) coloration, evaluated by absorbances of OD_550_, OD_420_, and OD_650_, was recorded to measure the activity of β-galactosidase (DU530 UV/Vis spectrophotometer, Beckman Coulter, CA, USA). All samples were tested in three duplicates and assays were performed at least three times.

### Quantitative real-time PCR assay.

Overnight cultured bacterial cells were diluted (1:50) in fresh KB medium and grown to the OD_600_ of 0.25, then 4 ml cultures were harvested by centrifugation at 4°C. After total RNA extraction (Iso plus, TaKaRa, Dalian, China) and cDNA preparation (PrimeScript RT reagent kit with gDNA Eraser, TaKaRa, Dalian, China), qRT-PCR were performed using the SYBR Premix *Ex Taq* (TaKaRa, Dalian, China) and CFX96 real-time PCR detection system (Bio-Rad, Hercules, CA, USA), all according to the manufacturer’s instruction. PCRs were set up using equivalent amounts of cDNA derived from the same amount of input RNA. The concentrations of all nucleic acid samples were measured using a NanoDrop 2000 spectrophotometer (Thermo Fisher Scientific, Inc., USA) and the 260/280 ratio of samples is limited between 1.8 and 2.0. At least three biological replicates were performed. As described previously, 2^-ΔCt^ method was used to analyze relative expression levels of target genes and the expression level of 16S in each individual sample was measured as the internal control. The equation used in our study is

$$\mathrm{Relative}\;\mathrm{expression}\;\mathrm{level}=2^{-(C_{t,X}-C_{t,R})}\times 10^{4}$$

where $C_{t,X}\;$ is the threshold cycle for target amplification and $C_{t,R}$ is the threshold cycle for reference (16S) amplification.

### Protein expression and purification.

The RstA gene was amplified and cloned into pMAL-c2X expression vector containing an additional maltose-binding protein (MBP) tag. The RstA bearing D52A mutation expression plasmid was obtained via overlap PCR. All these recombinant plasmids and the untreated pMAL-c2X were transformed to E. coli BL21(DE3) cells, which were developed in LB medium at 37°C supplemented with 50 μg/ml ampicillin. The protein expression was induced by 0.2 mM IPTG when OD_600_ reached 0.6 and cultured at 16°C for another 20 h. After harvested by centrifugation at 5,000 g for 10 min, the cells were resuspended in a column binding buffer containing 20 mM Tris–HCl at pH 8.0, 200 mM NaCl, 1 mM EDTA, 1 mM DTT, and lysed by sonication. The debris was removed via centrifugation at 12,000 g for 30 min and subsequently the supernatant was loaded onto an amylose resin affinity column (New England Biolabs, Inc., USA) for purification of the MBP protein or the MBP-tagged proteins, which were eluted by the binding buffer supplemented with 10 mM maltose. The purified proteins, MBP-RstA, MBP-D52A, and MBP, were concentrated and stored at –80°C for further usage.

### Protein phosphorylation *in vitro*.

The proteins were phosphorylated *in vitro* as a standard reaction reported before (56). In brief, the protein was diluted into the buffer containing 100 mM Tris-HCl (pH 7.4), 10 mM MgCl_2_, 125 mM KCl, and 50 mM acetyl phosphate (lithium potassium acetyl phosphate, sigma) with a final concentration of 100 μg/ml and incubated for a further 1 h at 30°C. Then the buffer was replaced with 20 mM Tris-HCl (pH 7.4) by a centrifugal filter (Amicon Ultra-0.5 Centrifugal Filter Unit with Ultracel-3 membrane, Millipore). As for control groups, the protein was processed with the same method except acetyl phosphate was removed from the dilution buffer.

### Electrophoretic mobility shift assay and DNase I footprinting assay.

Promoters of *emhA* and *emhC*, designated as FAM-*emhA* and FAM-*mexC*, were amplified and purified for EMSA and DNase I footprinting assays. For EMSAs, 50 ng of purified promoters were incubated with different amounts of proteins for 30 min at room temperature. In a total volume of 20 μl, the reaction mixture contained 50 mM Tris-HCl at pH 7.5, 10 mM MgCl_2_, 10% (vol/vol) glycerol, 0.5 mM EDTA, 50 mM KCl, and 3 μM bovine serum albumin (BSA) to prevent unspecific binding (54). Separated by 6% native PAGE, electrophoretic bands were detected by a UVP BioSpectrum Imaging system (UVP, CA, USA). For DNase I footprinting assays, 3 pmol DNA was incubated with different concentrations of proteins for 30 min at room temperature as well, followed by digestion with DNase I (Promega) for 2.5 min at 30°C. The reactions were quenched by adding the stop-buffer consisting of 0.15% SDS, 200 mM sodium acetate, and 30 mM EDTA, and then further incubated in a water bath at 70°C for 30 min. The digestion production was extracted with isopropanol/chloroform and precipitated with ethanol and the pellets were dissolved in 30 μl Milli-Q water.

### Sample preparation and LC-MS/MS analysis.

All proteomics samples were prepared using the modified protocol which had been reported by Matthias Mann et al. (57). Briefly, bacterial cells of the wild-type, *ΔrstA*, and *rstA^D52A^* strains from early stationary phase (OD_600_ = 0.8) were collected and resuspended in 6 M Urea, 100 mM DTT, and 100 mM Tris-HCl (pH 7.0), and proteins were digested with trypsin overnight. The total peptide was desalted with C18-column and dissolved into 0.1% formic acid (FA) at a final concentration of 100 ng/μl. For MS analysis, 1 μg peptide of each sample was subjected to nanoflow liquid chromatography-tandem mass spectrometry analysis on an Orbitrap Fusion Lumos mass spectrometer (Thermo Scientific) coupled online to an EASY-nLC 1200 system in the data-dependent mode. Using a 150 mm × 75 μm C18 column with 2 μm particles, peptides were separated on a 90-min nonlinear gradient: 5%–35% buffer B for 60 min, 35%–80% buffer B for 20 min, and 100% buffer B for 10 min (buffer A: 0.1% FA; buffer B: 80% acetonitrile, 0.1% FA) at 300 nL/min constant flow rate. Source voltage and current were set to 2.5 KV and 100 A, respectively. All MS measurements were performed in the positive ion mode and acquired across the mass range of 300–1,800 *m/z*. Raw mass spectrometry files were analyzed by ProteinDiscovery (version 2.3) (58) and MS/MS spectra was searched against the full protein sequences of P. fluorescens 2P24 with the fixed modification of cysteine carbamidomethylation, and variable modification methionine oxidation configured. Other parameters were set up using the default values and the false discovery rate (FDR) was set to 0.01 for both peptide and protein identifications. Further bioinformatics and statistical analyses were performed using the software Perseus (59).

### Data availability.

The MS raw files and proteome sequences of P. fluorescens 2P24 have been deposited to the ProteomeXchange Consortium via the PRIDE partner repository with the data set identifier PXD018793 (60).

10.1128/mSystems.00911-21.4 TABLE S4 Up- and down-regulated proteins in the *rstA* deletion strain. Download Table S4, DOCX file, 0.03 MB. Copyright © 2021 Li et al. 2021 Li et al. https://creativecommons.org/licenses/by/4.0/ This content is distributed under the terms of the Creative Commons Attribution 4.0 International license.
