# Supplementary material for: The Two-Component System RstA/RstB Regulates Expression of Multiple Efflux Pumps and Influences Anaerobic Nitrate Respiration in Pseudomonas fluorescens
Source: mSystems. 2021 Nov 2;6(6):e00911-21. doi: 10.1128/mSystems.00911-21 (PMC8562477; doi:10.1128/mSystems.00911-21)
Supplement: TABLE S2 [file msystems.00911-21-st002.docx]

**Supplementary Table S2** Primers used in this study.

| Primers for expression in *E. coli* BL21(DE3) | DNA | Sequences (5’ to 3’) targets |
| --- | --- | --- |
| RstA-F | *rstA* | CGCGGATCCATGCCCAACATCCTTCTGGTC |
| RstA-R |  | CCAATGCATTGGTTCTGCAGTCAGTATTCCCACTCGGAACG |
| D52A-F | *rstA^D52A^* | CCTGGTGATCCTCGCCCTGATGCTGCCG |
| D52A-R |  | CCCGGCAGCATCAGGGCGAGGATCACCA |
| Primers for EMSA assays |  |  |
| FAM-P*emhA*-F | *emhA* | GGCAATAAACACCTCAATCAGG |
| FAM-P*emhA*-R |  | AAATCCTCGGGTCCAGGC |
| FAM-P*mexC*-F | *mexC* | CGGCCTGTGTGATTACACAATTT |
| FAM-P*mexC*-R |  | GGTGCTTCCCCAATATTGAGC |
| Primers for *lacZ* reporter |  |  |
| pRG970-P*emhA* -F | *emhA* | ATTCCCTGACTGGATGGGCCCCTAGGGGCAATAAACACCTCAATCAGGATG |
| pRG970-P*emhA* -R |  | CCATGGAGATCTTCTTCGAACGGAAATCCTCGGGTCCAGGC |
| Primers for mutants construction |  |  |
| L∆*rstA*-F | *rstA* | AAACAGCTATGACATGATTACGAATTCAACCGATGTATGTCACCTACGAAGCG |
| L∆*rstA*-R |  | GCTGAGCACGCTGACCTGATAGC |
| R∆*rstA*-F |  | CAGGTCAGCGTGCTCAGCAAGC |
| R∆*rstA*-R |  | GTTGTAAAACGACGGCCAGTGCCAAGCTTATCGATCACCACGTCCGGTGC |
| L∆*rstBS*-F | *rstrstBS* | AAACAGCTATGACATGATTACGAATTCATATTCGGATCGGGTTGGTGGT |
| L∆*rstBS*-R |  | CACTAACTCCGGCATCAAGTAGATC |
| R∆*rstBS*-F |  | ATCTACTTGATGCCGGAGTTAGTGAGTTGGCTGTATTGGAGCATCAAC |
| R∆*rstBS*-R |  | TAAAACGACGGCCAGTGCCAAGCTTAAGATCGTGCTGGCGCAATC |
| L∆*rstBK*-F | *rstrstBK* | AAACAGCTATGACATGATTACGAATTCAGTTACCTGGAACGCAACGGC |
| L∆*rstBK*-R |  | TAGCCAAAGCAACAGGCAGGC |
| R∆*rstBK*-F |  | CCTGCCTGTTGCTTTGGCTAGGCATGCTCAGTTACCCAAACAA |
| R∆*rstBK*-R |  | TAAAACGACGGCCAGTGCCAAGCTTCTGCAACCCGGACATCGACA |
| L∆*mexC*-F | *mexC* | AAACAGCTATGACATGATTACGAATTCACGATGAACTGGGCCAACTGGC |
| L∆*mexC*-R |  | TCAGCTCGTTGCTGATCGACAGTG |
| R∆*mexC*-F |  | GTCGATCAGCAACGAGCTGAGACGCCGAGCAGAAGGTCAGC |
| R∆*mexC*-R |  | TAAAACGACGGCCAGTGCCAAGCTTTGACGAACGGCGACGTGTCG |
| L∆*bcr*-F | *bcr* | AAACAGCTATGACATGATTACGAATTCGTGAATGACTACAGGATCAACCTTGC |
| L∆*bcr*-R |  | CAGGGTCAGTTGGATGTGCTTTT |
| R∆*bcrC*-F |  | GAAAAGCACATCCAACTGACCCTGCAGGGATGTCTGCAGTTTTCCG |
| R∆*bcrC*-R |  | TAAAACGACGGCCAGTGCCAAGCTTGGAATTGTTGTTCGAGCGCCT |
| M*rstA*-D52A-F | *rstA^D52A^* | CCTGGTGATCCTCGCCCTGATGCTGCCG |
| M*rstA*-D52A-R |  | CCCGGCAGCATCAGGGCGAGGATCACCA |
| M*rstA*-D52E-F | *rstA^D52E^* | TGGTGATCCTCGAACTGATGCTG |
| M*rstA*-D52E-R |  | CGGCAGCATCAGTTCGAGGAT |
| Primers for qPCR |  |  |
| *emhA-*F | *emhA* | TCGCTGAAGTTCGCCCACA |
| *emhA-*R |  | CGGCGGTGTCGTATTCCTG |
| *emhC-*F | *emhC* | TGCCTCCAACCAGGACTACTACC |
| *emhC-*R |  | CTTCGTCTTTCACTGGTTCGTTC |
| *mexC-*F | *mexC* | ACGACGGCAGCCCTTACCC |
| *mexC-*R |  | GGCGTCCAGCAGCAACACC |
| *bcr-*F | *bcr* | TTACTTCCTCGGCTTATCCAT |
| *bcr-*R |  | CATCAACTGCGAAAACACCTT |
| *16s-F* | *16s* | AAGCCTGATCCAGCCATGCC |
| *16s-R* |  | TAACGAACCACCTACGCGCG |
