# Supplementary material for: The Two-Component System RstA/RstB Regulates Expression of Multiple Efflux Pumps and Influences Anaerobic Nitrate Respiration in Pseudomonas fluorescens
Source: mSystems. 2021 Nov 2;6(6):e00911-21. doi: 10.1128/mSystems.00911-21 (PMC8562477; doi:10.1128/mSystems.00911-21)
Supplement: TABLE S3 [file msystems.00911-21-st003.docx]

**Supplementary Table S3** The genes in *P. fluorescens* FW300-N2E2 identified by cofitness and their respective orthologues in *P. fluorescens* 2P24

| ***P . fluorescens* FW300-N2E2** | ***P . fluorescens* 2P24** |
| --- | --- |
| *rstA（Pf6N2E2_463）* | *rstA（PFLP_02434）* |
| *QseC（Pf6N2E2_464）* | *rstB（PFLP_02435）* |
| *CmeA（Pf6N2E2_1660）* | *mexC（**PFLP_03468）* |
| *CmeB（Pf6N2E2_1661）* | *mexD（PFLP_03469）* |
| *MFS（Pf6N2E2_3484）* | *bcr （**PFLP_00760）* |
