# Supplementary figures and images for: The Two-Component System RstA/RstB Regulates Expression of Multiple Efflux Pumps and Influences Anaerobic Nitrate Respiration in Pseudomonas fluorescens
Source: mSystems. 2021 Nov 2;6(6):e00911-21. doi: 10.1128/mSystems.00911-21 (PMC8562477; doi:10.1128/mSystems.00911-21)

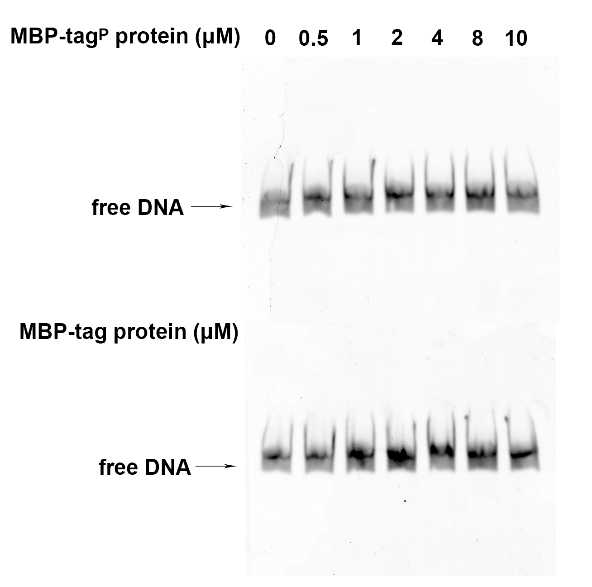

Supplement: FIG S1 [file msystems.00911-21-sf001.tif]

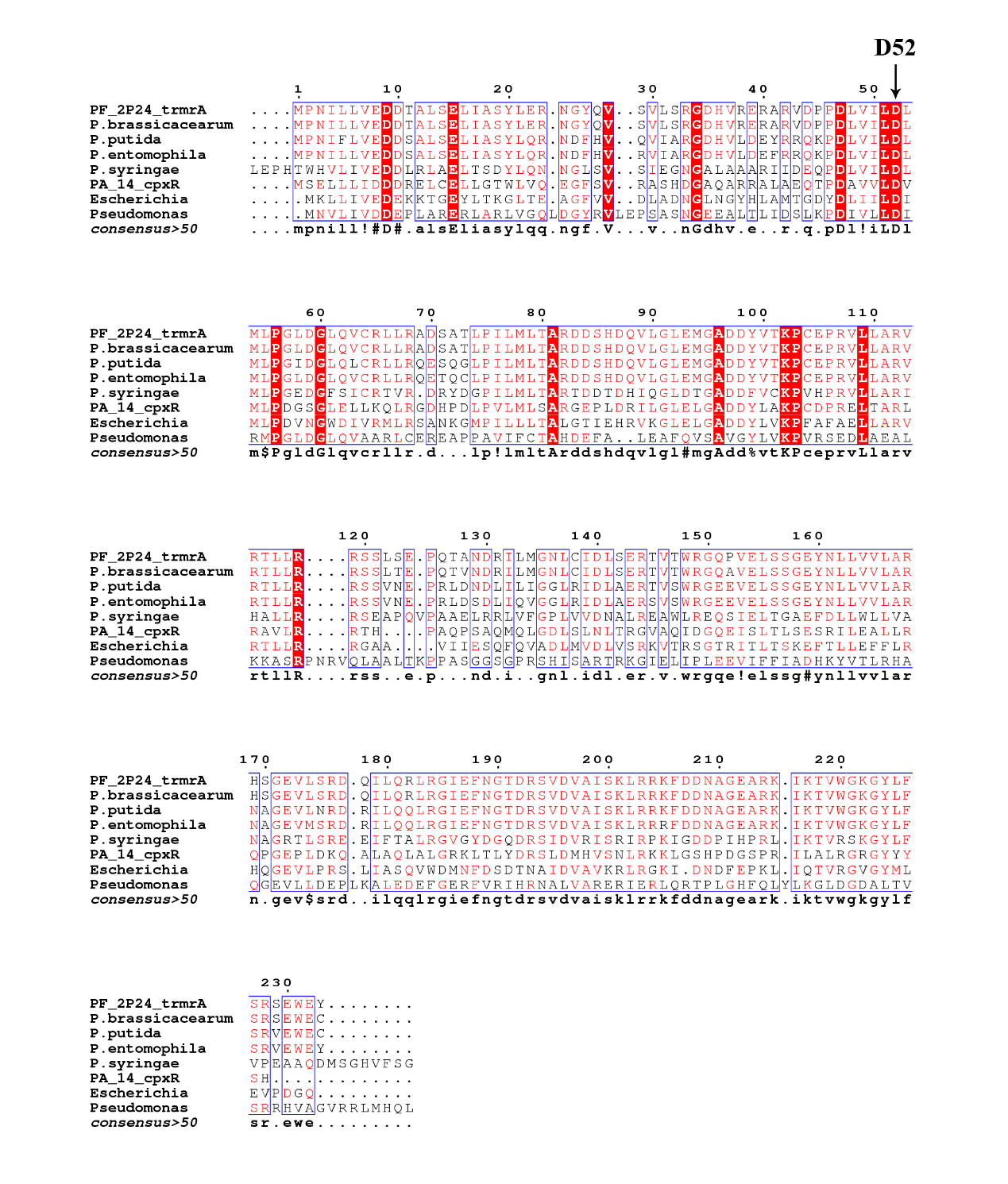

Supplement: FIG S2 [file msystems.00911-21-sf002.tif]

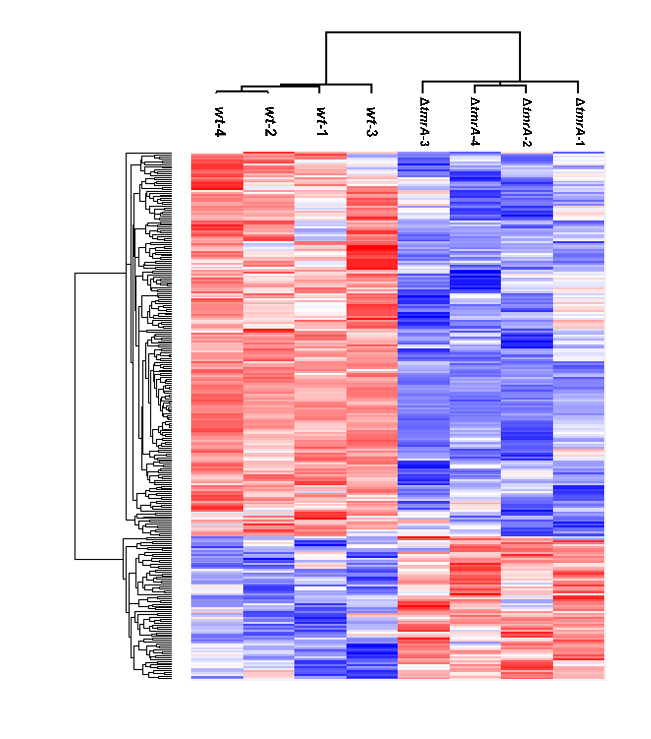

Supplement: FIG S3 [file msystems.00911-21-sf003.tif]
