# Supplementary material for: The Two-Component System RstA/RstB Regulates Expression of Multiple Efflux Pumps and Influences Anaerobic Nitrate Respiration in Pseudomonas fluorescens
Source: mSystems. 2021 Nov 2;6(6):e00911-21. doi: 10.1128/mSystems.00911-21 (PMC8562477; doi:10.1128/mSystems.00911-21)
Supplement: TABLE S1 [file msystems.00911-21-st001.docx]

**Supplementary Table S1** Strains and plasmids used in this study.

| Strains or plasmid | Description | Source |
| --- | --- | --- |
| Strains  *E.coli* DH5α | F^–^ *endA1* *glnV44* *thi-1* *recA1* *relA1* *gyrA96* *deoR* *nupG* *purB20* φ80d*lacZ*ΔM15 Δ(*lacZYA-argF*)U169, hsdR17(*r_K_*^–^*m_K_*^+^), λ^–^ | Novagen |
| BL21 (DE3)  WM3064  *P. fluorescens* 2P24 | F– *ompT gal dcm lon hsdSB(rB^–^mB^–^)* λ(DE3 [*lacI lacUV5-T7p07 ind1 sam7 nin5*]) [*malB^+^*]_K-12_(λS)  *thrB1004 pro thi rpsl hsdS lacZ*ΔM15 RP4-1360 *Δ(araBAD)567 ΔdapA1341::[erm pir]*  Wild type; Ap^r^ | Novagen  This lab  (1) |
| Δ*rstA*  Δ*rstBkinase*  Δ*rstBsensor*  Δ*emhABC*  Δ*cmeA*  Δ*mfs*  *rstA^D52A^*  *rstA^D52E^*  Plasmids  PMAL-c2X  PMAL-c2X*-rstA*  PMAL-c2X*-rstA^D52A^* PMAL-c2X*-rstA^D52E^*  pRG970Km  pRG970Km-P*emhA*  pK18mobsacB  pK18-∆*rstA*  pK18-Δ*rstBkinase*  pK18- ∆*rstBsensor*  pK18- ∆*emhABC*  pK18- ∆*cmeA*  pK18- ∆*mfs*  pK18- *rstA^D52A^*  pK18- *rstA^D52E^* | *rstA* gene in-frame deletion in strain 2P24; Ap^r^  The kinase domain of *rstB* gene in-frame deletion in strain 2P24; Ap^r^  The sensor domain of *rstB* gene in-frame deletion in strain 2p24;Ap^r^  *emhABC* gene mutant; in-frame deletion in strain 2P24;Ap^r^  *cmeA* gene mutant; in-frame deletion in strain 2P24;Ap^r^  *mfs* gene mutant; in-frame deletion in strain 2P24;Ap^r^  the D52 of RstA was mutated to alanine in strain 2P24; Ap^r^  the D52 of RstA was mutated to glutamic acid in strain 2P24; Ap^r^  Expression vector; Ap^r^  Plasmid for overexpression of RstA; Ap^r^  Plasmid for overexpression of RstA^D52A^; Ap^r^  Plasmid for overexpression of RstA^D52E^; Ap^r^  Cloning vector for construction of transcriptional fusion in *P. fluorescens*; Km^r^  pRG970Km containing a *emhA*-*lacZ* transcriptional fusion; Km^r^  Suicide vector for generation of gene in-frame deletions, *sacB*, Km^r^  Vector for *rstA* gene deletion. Uses Pk18 backbone  Vector for *rstBkinase* gene deletion. Uses Pk18 backbone  Vector for *rstBsensor* gene deletion. Uses Pk18 backbone  Vector for *emhABC* gene deletion. Uses Pk18 backbone  Vector for *cmeA* gene deletion. Uses Pk18 backbone  Vector for *mfs* gene deletion. Uses Pk18 backbone  Vector for D52A site mutation of *rstA.* Uses Pk18 backbone  Vector for D52E site mutation of *rstA.* Uses Pk18 backbone | This study  This study  This study  This study  This study  This study  This study  This study  Addgene  This study  This study  This study  Addgene  (1)  This study  (2)  This study  This study  This study  This study  This study  This study  This study  This study |

1. Wei, H.L. and Zhang, L.Q. (2006) Quorum-sensing system influences root colonization and biological control ability in Pseudomonas fluorescens 2P24. Antonie Van Leeuwenhoek, 89, 267-280.

2. Schafer, A., Tauch, A., Jager, W., Kalinowski, J., Thierbach, G. and Puhler, A. (1994) Small mobilizable multi-purpose cloning vectors derived from the Escherichia coli plasmids pK18 and pK19: selection of defined deletions in the chromosome of Corynebacterium glutamicum. Gene, 145, 69-73.
