# Supplementary material for: The Two-Component System RstA/RstB Regulates Expression of Multiple Efflux Pumps and Influences Anaerobic Nitrate Respiration in Pseudomonas fluorescens
Source: mSystems. 2021 Nov 2;6(6):e00911-21. doi: 10.1128/mSystems.00911-21 (PMC8562477; doi:10.1128/mSystems.00911-21)
Supplement: TABLE S4 [file msystems.00911-21-st004.docx]

**Supplementary Table S4** Up- and down-regulated proteins in the *rstA* deletion strain.

| p-value | log2 (Fold Change) | IDs | | Description |  |
| --- | --- | --- | --- | --- | --- |
| Up-regulated |  |  | |  |  |
| 0.00199637 | 0.930244 | PFLP_00005 | | Gamma-aminobutyrate: proton symporter, AAT family |  |
| 0.0293771 | 0.618004 | PFLP_00011 | | GlyQ, glycyl-tRNA synthetase alpha chain |  |
| 0.00153734 | 0.997471 | PFLP_00202 | | GcdH, glutaryl-CoA dehydrogenase |  |
| 9.75E-05 | 0.932243 | PFLP_00203 | | Crotonobetainyl-CoA, carnitine CoA-transferase CaiB |  |
| 0.0133064 | 0.85512 | PFLP_00409 | | Septal ring factor EnvC, activator of murein hydrolases AmiA and AmiB |  |
| 0.0468115 | 0.702666 | PFLP_00652 | | Uncharacterized conserved protein, Alpha-E superfamily |  |
| 0.0264335 | 1.22566 | PFLP_01404 | | Transcriptional regulator, TetR family |  |
| 0.0034938 | 1.61888 | PFLP_01647 | | Flagellar biosynthesis protein FlhF |  |
| 0.0168142 | 0.615663 | PFLP_01702 | | Methylthioribose-1-phosphate isomerase |  |
| 0.0470434 | 0.825284 | PFLP_01934 | | Fic family protein |  |
| 0.0102574 | 0.743999 | PFLP_01972 | | DusA tRNA-U16, U17-dihydrouridine synthase |  |
| 0.0373891 | 1.66228 | PFLP_01988 | | Hypothetical protein |  |
| 0.0287062 | 0.834288 | PFLP_02119 | | L-glutamine synthetase |  |
| 0.0245219 | 0.665261 | PFLP_02132 | | Hypothetical protein |  |
| 0.0103731 | 0.861398 | PFLP_02278 | | NAD(P)H dehydrogenase (quinone) |  |
| 0.00142847 | 0.715338 | PFLP_02334 | | Major Facilitator Superfamily protein |  |
| 0.00164186 | 1.23634 | PFLP_02336 | | 3-oxoacyl-[acyl-carrier protein] reductase |  |
| 0.0231134 | 0.712324 | PFLP_02400 | | Predicted transcriptional regulator YdeE, contains AraC-type DNA-binding domain |  |
| 0.0383859 | 0.706105 | PFLP_02916 | | Short-chain dehydrogenase involved in D-alanine esterification of teichoic acids |  |
| 0.000388851 | 1.86313 | PFLP_03349 | | Beta-barrel assembly machine subunit BamE |  |
| 0.00226078 | 1.94966 | PFLP_03388 | | Polyketide cyclase/dehydrase and lipid transport |  |
| 0.00044754 | 1.81975 | PFLP_03482 | | 3-Oxoacyl-[acyl-carrier-protein (ACP)] synthase III |  |
| 0.016214 | 2.20041 | PFLP_03614 | | Amidase |  |
| 0.0124761 | 1.63033 | PFLP_03855 | | Quinate dehydrogenase (quinone) |  |
| 0.0271294 | 1.04541 | PFLP_03998 | | Hypothetical protein |  |
| 0.01512 | 0.618759 | PFLP_04050 | | Surfactin synthase thioesterase subunit |  |
| 0.00435501 | 0.857864 | PFLP_04540 | | Aromatic amino acid: proton symporter, AAT family |  |
| 0.0310896 | 1.74707 | PFLP_04607 | | Two-component system, response regulator AauR |  |
| 2.08E-05 | 2.01689 | PFLP_04700 | | Methionine synthase (B12-independent) |  |
| 0.0484494 | 1.69856 | PFLP_05030 | | Protein of unknown function (DUF4124) |  |
| 0.0467443 | 1.06402 | PFLP_05226 | | Rare lipoprotein A |  |
| 0.0108125 | 1.42344 | PFLP_05299 | | RibH, 6,7-dimethyl-8-ribityllumazine synthase |  |
| 0.00128279 | 0.672854 | PFLP_05892 | | Type VI secretion system protein ImpA |  |
| Down-regulated |  | |  |  | |
| 0.0180916 | -0.889209 | | PFLP_00041 | Thiol: disulfide interchange protein DsbA | |
| 0.00959665 | -0.643455 | | PFLP_00100 | Protein SCO1/2 | |
| 0.00652905 | -1.84109 | | PFLP_00136 | Hypothetical protein | |
| 0.000950448 | -1.90449 | | PFLP_00137 | PotF, putrescine transport system substrate-binding protein | |
| 0.011738 | -1.72558 | | PFLP_00178 | HupA, bacterial nucleoid protein HU alpha subunit | |
| 0.00103223 | -0.762781 | | PFLP_00197 | Succinyl-CoA: acetate CoA-transferase | |
| 0.0114384 | -2.20214 | | PFLP_00463 | RNA methyltransferase, RsmE family | |
| 5.09E-06 | -2.2453 | | PFLP_00522 | FabF 3-oxoacyl-[acyl-carrier-protein] synthase II | |
| 0.0339789 | -1.25547 | | PFLP_00631 | HflX GTP-binding protein HflX | |
| 0.0319461 | -1.1059 | | PFLP_00658 | Azurin | |
| 0.000218207 | -1.09815 | | PFLP_00705 | Iron complex outer membrane receptor protein | |
| 8.53E-06 | -1.72344 | | PFLP_00707 | Hypothetical protein | |
| 0.00868234 | -1.08127 | | PFLP_00900 | Zein-binding | |
| 0.0205052 | -1.62826 | | PFLP_00918 | OpuCA, osmoprotectant transport system ATP-binding protein | |
| 0.000617785 | -1.36125 | | PFLP_00934 | Predicted secreted protein | |
| 0.0230901 | -0.820655 | | PFLP_00952 | GatC, aspartyl/glutamyl-tRNA (Asn/Gln) amidotransferase subunit C | |
| 0.0473699 | -0.921669 | | PFLP_00969 | Superoxide dismutase, Fe-Mn family | |
| 0.00725303 | -1.45864 | | PFLP_01098 | XenA, 2,4-dienoyl-CoA reductase | |
| 0.0429089 | -0.850781 | | PFLP_01139 | Protein of unknown function (DUF4197) | |
| 0.0257584 | -1.1514 | | PFLP_01287 | Hypothetical protein | |
| 1.52E-05 | -0.806553 | | PFLP_01312 | ArcC, carbamate kinase | |
| 0.000105192 | -1.10624 | | PFLP_01313 | ArgF, ornithine carbamoyltransferase | |
| 0.00843181 | -0.700225 | | PFLP_01314 | ArcA, arginine deiminase | |
| 7.02E-05 | -1.05253 | | PFLP_01426 | Alcohol dehydrogenase | |
| 0.0403601 | -0.92231 | | PFLP_01485 | Methyl-accepting chemotaxis protein | |
| 0.0353561 | -1.8181 | | PFLP_01517 | Glutamine amidotransferase | |
| 0.000114985 | -1.79585 | | PFLP_01519 | Predicted arabinose efflux permease, MFS family | |
| 0.000460914 | -1.79734 | | PFLP_01520 | UPF0271 protein | |
| 0.00149825 | -1.09952 | | PFLP_01521 | Sensor histidine kinase inhibitor, KipI family | |
| 3.10E-05 | -1.64776 | | PFLP_01522 | Biotin-dependent carboxylase uncharacterized domain-containing protein | |
| 0.0379786 | -0.904029 | | PFLP_01651 | CheZ, chemotaxis phosphatase | |
| 0.0236389 | -0.728354 | | PFLP_01666 | CcmE, cytochrome c-type biogenesis protein | |
| 0.0038506 | -1.05155 | | PFLP_01670 | Cytochrome c-type biogenesis protein CcmH | |
| 0.0287525 | -1.95189 | | PFLP_01748 | Putative ABC transport system ATP-binding protein | |
| 0.000103754 | -1.32741 | | PFLP_01820 | Amino acid adenylation domain-containing protein | |
| 0.00382286 | -0.852309 | | PFLP_01821 | Non-ribosomal peptide synthase domain TIGR01720/amino acid adenylation domain-containing protein | |
| 0.00511285 | -1.10483 | | PFLP_01830 | Diaminobutyrate aminotransferase apoenzyme | |
| 0.000743701 | -1.16473 | | PFLP_01879 | Rmf, ribosome modulation factor | |
| 0.00533243 | -1.21872 | | PFLP_01907 | Oxygen-independent coproporphyrinogen-3 oxidase | |
| 0.0291323 | -0.751681 | | PFLP_01910 | Cu2+-exporting ATPase | |
| 0.023597 | -0.854003 | | PFLP_01920 | Cytochrome c oxidase cbb3-type subunit III | |
| 0.000999053 | -2.22673 | | PFLP_01921 | Cytochrome c oxidase cbb3-type subunit 4 | |
| 0.00507685 | -1.19355 | | PFLP_01922 | Cytochrome c oxidase cbb3-type subunit 2 | |
| 0.00187638 | -1.09725 | | PFLP_01923 | CcoN-1, cytochrome c oxidase cbb3-type subunit 1 | |
| 0.00915074 | -1.11553 | | PFLP_01960 | LolD, lipoprotein-releasing system ATP-binding protein | |
| 0.000153073 | -1.18164 | | PFLP_02042 | Hypothetical protein | |
| 0.0315945 | -1.1349 | | PFLP_02116 | Arginine succinyltransferase | |
| 0.0158137 | -0.659511 | | PFLP_02144 | Porphobilinogen synthase | |
| 0.02781 | -0.712217 | | PFLP_02164 | DNA-binding transcriptional regulator, MocR family, contains an aminotransferase domain | |
| 0.0414086 | -1.80476 | | PFLP_02171 | 1,4-alpha-glucan branching enzyme | |
| 2.14E-06 | -1.60787 | | PFLP_02222 | Putative two-component system protein, hydrogenase maturation factor HypX/HoxX | |
| 0.0492198 | -0.78035 | | PFLP_02226 | Uncharacterized conserved protein, DUF934 family | |
| 0.00218296 | -1.17767 | | PFLP_02256 | BdhA, 3-hydroxybutyrate dehydrogenase | |
| 0.0250466 | -0.659102 | | PFLP_02381 | Purine-binding chemotaxis protein CheW | |
| 0.00361051 | -0.675681 | | PFLP_02382 | Methyl-accepting chemotaxis protein | |
| 0.0492165 | -0.864766 | | PFLP_02385 | CheY-1, two-component system, chemotaxis family, response regulator CheY | |
| 0.0417146 | -1.12853 | | PFLP_02388 | Sugar-phosphatase | |
| 1.16E-06 | -1.06752 | | PFLP_02476 | Hydrogen cyanide synthase HcnB | |
| 0.00374046 | -1.15725 | | PFLP_02477 | Hydrogen cyanide synthase HcnC | |
| 0.00729461 | -1.16037 | | PFLP_02552 | Nucleotide-binding universal stress protein, UspA family | |
| 0.0232665 | -1.22473 | | PFLP_02570 | Hydroxyacylglutathione hydrolase | |
| 0.0414921 | -1.41115 | | PFLP_02692 | Cytochrome c, mono- and diheme variants | |
| 0.000338696 | -1.63195 | | PFLP_02693 | Uroporphyrin-III C-methyltransferase | |
| 0.0119108 | -1.02904 | | PFLP_02696 | Transcriptional regulator, AsnC family | |
| 0.019736 | -0.909881 | | PFLP_02699 | Protein NirF | |
| 0.000752253 | -1.03229 | | PFLP_02716 | Pyridoxamine 5'-phosphate oxidase | |
| 0.006352 | -1.27321 | | PFLP_02717 | Molybdenum cofactor biosynthesis protein B | |
| 0.00688798 | -1.3699 | | PFLP_02719 | Putative protease | |
| 0.00355659 | -1.33166 | | PFLP_02722 | cAMP-binding domain of CRP or a regulatory subunit of cAMP-dependent protein kinases | |
| 1.15E-06 | -2.92207 | | PFLP_02727 | MFS transporter, NNP family, nitrate/nitrite transporter | |
| 3.43E-05 | -2.93498 | | PFLP_02728 | Respiratory nitrate reductase, alpha subunit | |
| 3.01E-06 | -3.53082 | | PFLP_02729 | Respiratory nitrate reductase, alpha subunit | |
| 0.000294004 | -2.75611 | | PFLP_02730 | NarH respiratory nitrate reductase beta subunit | |
| 0.0118292 | -3.94507 | | PFLP_02731 | Respiratory nitrate reductase chaperone NarJ | |
| 0.000941529 | -2.15574 | | PFLP_02732 | Respiratory nitrate reductase gamma subunit | |
| 0.000556003 | -4.53393 | | PFLP_02733 | Peptidyl-prolyl cis-trans isomerase C | |
| 0.00194378 | -1.47978 | | PFLP_02735 | Ribonucleoside-triphosphate reductase class III catalytic subunit | |
| 0.00808529 | -1.8357 | | PFLP_02738 | Methyl-accepting chemotaxis protein | |
| 0.0202439 | -1.44467 | | PFLP_02849 | Beta-hydroxylase | |
| 0.0293943 | -0.673873 | | PFLP_02850 | Bifunctional non-homologous end joining protein LigD | |
| 6.52E-05 | -2.84399 | | PFLP_02877 | OprN, outer membrane protein, multidrug efflux system | |
| 0.0021636 | -1.17349 | | PFLP_02879 | Multidrug efflux pump | |
| 0.0114904 | -1.25224 | | PFLP_02880 | Membrane fusion protein, multidrug efflux system | |
| 0.00259453 | -2.14449 | | PFLP_03026 | Outer membrane porin, OprD family | |
| 0.00379921 | -1.42977 | | PFLP_03027 | Predicted arabinose efflux permease, MFS family | |
| 8.61E-05 | -2.03634 | | PFLP_03029 | Uncharacterized protein YcsI, UPF0317 family | |
| 2.37E-05 | -2.28606 | | PFLP_03030 | Hypothetical protein | |
| 0.0203068 | -2.6037 | | PFLP_03031 | DNA-binding transcriptional regulator, LysR family | |
| 0.0498641 | -1.19132 | | PFLP_03047 | CDP-diacylglycerol-serine O-phosphatidyltransferase | |
| 0.0187661 | -1.64033 | | PFLP_03191 | Uncharacterized protein YjbI, contains pentapeptide repeats | |
| 0.0122773 | -0.810584 | | PFLP_03305 | Heat shock protein Hsp20 | |
| 0.00013295 | -1.29032 | | PFLP_03307 | Universal stress protein E | |
| 0.0117387 | -1.0336 | | PFLP_03308 | Xylulose-5-phosphate/fructose-6-phosphate phosphoketolase | |
| 0.00184849 | -1.04967 | | PFLP_03313 | Universal stress protein E | |
| 4.90E-05 | -1.27098 | | PFLP_03317 | Universal stress protein E | |
| 0.00518137 | -2.17607 | | PFLP_03469 | Multidrug efflux pump | |
| 0.0266895 | -1.64262 | | PFLP_03616 | Rieske [2Fe-2S] domain-containing protein | |
| 0.0266522 | -1.37109 | | PFLP_03617 | Nucleoside-diphosphate-sugar epimerase | |
| 0.00166432 | -1.48327 | | PFLP_03618 | Crotonobetaine/carnitine-CoA ligase | |
| 0.01858 | -1.82264 | | PFLP_03623 | Phosphopantetheine attachment site | |
| 0.000144982 | -2.06482 | | PFLP_03632 | FAD binding domain-containing protein | |
| 2.31E-05 | -1.52845 | | PFLP_03633 | Polyketide biosynthesis enoyl-CoA hydratase PksI | |
| 0.000808362 | -2.09772 | | PFLP_03636 | Hydroxymethylglutaryl-CoA synthase | |
| 0.0102494 | -2.10577 | | PFLP_03638 | Polyketide biosynthesis acyl carrier protein | |
| 0.000760909 | -1.29568 | | PFLP_03639 | Dihydroflavonol-4-reductase | |
| 0.0260256 | -0.993909 | | PFLP_03645 | Acyl transferase domain-containing protein | |
| 0.00124045 | -1.86955 | | PFLP_03647 | Acyl transferase domain-containing protein | |
| 9.02E-05 | -1.28438 | | PFLP_03648 | Trans-AT polyketide synthase, acyltransferase and oxidoreductase domain-containing protein | |
| 0.00108249 | -1.7758 | | PFLP_03649 | Acyl transferase domain-containing protein | |
| 0.00173573 | -1.24488 | | PFLP_03651 | Polyketide synthase PksL | |
| 0.00575326 | -1.18083 | | PFLP_03652 | Natural product biosynthesis luciferase-like monooxygenase domain-containing protein | |
| 0.0273476 | -0.616291 | | PFLP_03801 | Methyl-accepting chemotaxis protein | |
| 0.0266499 | -0.823277 | | PFLP_03933 | Carbohydrate ABC transporter substrate-binding protein, CUT1 family | |
| 5.48E-05 | -1.10926 | | PFLP_03984 | IbpA, molecular chaperone | |
| 0.0423865 | -1.53729 | | PFLP_04001 | Putative protease | |
| 0.00789834 | -1.0377 | | PFLP_04028 | L-ornithine N5-oxygenase | |
| 6.55E-05 | -1.46459 | | PFLP_04037 | Zn-dependent dipeptidase, dipeptidase homolog | |
| 0.0226046 | -1.62801 | | PFLP_04039 | Formylglycine-generating enzyme, required for sulfatase activity, contains SUMF1/FGE domain | |
| 0.00433549 | -0.895464 | | PFLP_04040 | Putative ATP-binding cassette transporter | |
| 0.00368676 | -0.861428 | | PFLP_04041 | Amino acid adenylation domain-containing protein | |
| 0.00149295 | -0.992944 | | PFLP_04043 | Amino acid adenylation domain-containing protein | |
| 0.00891895 | -0.799939 | | PFLP_04045 | Non-ribosomal peptide synthase domain TIGR01720/amino acid adenylation domain-containing protein | |
| 0.00117125 | -2.40056 | | PFLP_04046 | Amino acid adenylation domain-containing protein | |
| 0.000886302 | -0.841358 | | PFLP_04047 | Amino acid adenylation domain-containing protein | |
| 0.00975711 | -0.639588 | | PFLP_04048 | Non-ribosomal peptide synthase domain TIGR01720 | |
| 0.000187606 | -0.659696 | | PFLP_04087 | Hypothetical protein | |
| 0.0079381 | -1.10378 | | PFLP_04091 | Zinc transport system ATP-binding protein | |
| 0.00394024 | -0.953084 | | PFLP_04160 | Uncharacterized zinc-type alcohol dehydrogenase-like protein | |
| 0.00112177 | -1.09596 | | PFLP_04164 | 2-keto-3-deoxy-phosphogalactonate aldolase | |
| 1.90E-05 | -2.023 | | PFLP_04263 | ABC transporter substrate binding protein | |
| 0.0158903 | -0.918552 | | PFLP_04304 | Sbp2, sulfate transport system substrate-binding protein | |
| 0.00150818 | -0.997674 | | PFLP_04531 | Protein of unknown function (DUF2025) | |
| 0.0209955 | -1.27366 | | PFLP_04554 | CxxC, motif-containing protein, DUF1111 family | |
| 0.00952687 | -0.731998 | | PFLP_04645 | Starvation-inducible DNA-binding protein | |
| 0.0105299 | -0.888077 | | PFLP_04686 | KgtP MFS transporter, MHS family, alpha-ketoglutarate permease | |
| 0.00136134 | -0.773984 | | PFLP_04921 | MioC protein | |
| 0.0311208 | -2.01869 | | PFLP_04944 | AlgW serine protease DegS | |
| 0.0483193 | -0.881713 | | PFLP_04979 | Tetratricopeptide repeat-containing protein | |
| 0.000609209 | -1.69204 | | PFLP_05065 | Hypothetical protein | |
| 0.0127469 | -0.884338 | | PFLP_05068 | MOSC domain-containing protein YiiM | |
| 0.0412621 | -0.73382 | | PFLP_05135 | Catalase-related immune-responsive protein | |
| 8.70E-05 | -0.995529 | | PFLP_05136 | Catalase | |
| 0.0297353 | -1.75525 | | PFLP_05167 | Hypothetical protein | |
| 0.0298533 | -0.756037 | | PFLP_05189 | Hypothetical protein | |
| 0.00337405 | -1.80748 | | PFLP_05312 | Outer membrane protein | |
| 0.00813513 | -1.05803 | | PFLP_05358 | RpmC, LSU ribosomal protein L29P | |
| 0.0062381 | -0.644847 | | PFLP_05459 | PqqC, pyrroloquinoline-quinone synthase | |
| 0.0315537 | -0.690438 | | PFLP_05634 | Protein of unknown function (DUF4426) | |
| 0.0210539 | -1.78981 | | PFLP_05636 | Oxygen-independent coproporphyrinogen-3 oxidase | |
| 0.0107732 | -1.57681 | | PFLP_05762 | HlyD family secretion protein | |
| 0.0282997 | -0.953678 | | PFLP_05789 | Uncharacterized conserved protein YcfL | |
| 0.0466226 | -1.39723 | | PFLP_05976 | AtpB, F-type H+-transporting ATPase subunit a | |
| 0.0107975 | -0.91131 | | PFLP_05981 | MnmG, tRNA uridine 5-carboxymethylaminomethyl modification enzyme | |
